# Protective Effects of Dietary Antioxidants against Vanadium-Induced Toxicity: A Review

**DOI:** 10.1155/2020/1490316

**Published:** 2020-01-07

**Authors:** Iwona Zwolak

**Affiliations:** Laboratory of Oxidative Stress, Centre for Interdisciplinary Research, The John Paul II Catholic University of Lublin, Konstantynów 1 J, 20-708 Lublin, Poland

## Abstract

Vanadium (V) in its inorganic forms is a toxic metal and a potent environmental and occupational pollutant and has been reported to induce toxic effects in animals and people. *In vivo* and *in vitro* data show that high levels of reactive oxygen species are often implicated in vanadium deleterious effects. Since many dietary (exogenous) antioxidants are known to upregulate the intrinsic antioxidant system and ameliorate oxidative stress-related disorders, this review evaluates their effectiveness in the treatment of vanadium-induced toxicity. Collected data, mostly from animal studies, suggest that dietary antioxidants including ascorbic acid, vitamin E, polyphenols, phytosterols, and extracts from medicinal plants can bring a beneficial effect in vanadium toxicity. These findings show potential preventive effects of dietary antioxidants on vanadium-induced oxidative stress, DNA damage, neurotoxicity, testicular toxicity, and kidney damage. The relevant mechanistic insights of these events are discussed. In summary, the results of studies on the role of dietary antioxidants in vanadium toxicology appear encouraging enough to merit further investigations.

## 1. Introduction

It is well known that one of the mechanisms associated with the toxicity of heavy metals is oxidative stress [1] defined as an imbalance between the production of reactive oxygen species and antioxidant defenses, which may lead to tissue injury [2]. Currently, the preferred medical treatment of metal poisoning includes chelation therapy, which facilitates removal of the excess of the metal from soft tissues and excretion in urine. However, the serious side effects that may occur during the chelation therapy such as depletion of essential minerals, prooxidant effects, hepatic and renal toxicity, and no removal of heavy metals from intracellular compartments are the major drawbacks of this treatment [3]. Since oxidative stress plays a pivotal role in the pathogenesis of metal toxicity, the use of antioxidants as a supplementary therapy to conventional chelation treatment has been proposed [3, 4].

In literature, the beneficial action of dietary and plant-derived antioxidants on toxicity of some of the heavy metals has been reported. For example, flavonoids such as epigallocatechin gallate and curcumin have been shown to possess protective activity against cadmium-induced nephrotoxicity in rats [5, 6]. Catechin hydrate has been found to reduce cytotoxic and genotoxic effects of cadmium in human peripheral blood lymphocytes [7]. Sulforaphane (organosulfur compound) protected human mesenchymal stem cells against cadmium-induced changes in nuclear morphology, depletion of mitochondrial membrane potential, and alteration of gene expression [8]. The administration of epigallocatechin gallate attenuated arsenic-induced oxidative damage in the liver of rats [9]. An inhibitory effect of dietary supplement containing a mixture of grape seed extract, tea polyphenols, and probiotics on toxicity of lead to mice has also been reported [10]. A human study with car battery workers found that garlic supplementation significantly reduced signs of occupational chronic lead poisoning such as irritability, headache, and mean systolic blood pressure [11]. In the same study, garlic has also significantly decreased blood lead concentrations.

In this review, the focus is placed on studies evaluating the use of natural antioxidant compounds against vanadium toxicity. In industry, vanadium is a widely used transition metal, and the global demand and production of vanadium is on the increase [12]. This certainly raises concerns over the detrimental effects of vanadium excess on human and animal health. People who were reported to encounter vanadium toxicity are those occupationally exposed to vanadium as well as those living in areas with high vanadium content in the air or water (described in the next chapter). In view of the fact that oxidative stress is an underlying mechanism of vanadium-induced toxicity, natural compounds with antioxidant properties are gaining increasing attention as cheap and safe antidotes against vanadium. To this end, this review explores the past and current data on the effectiveness of antioxidants of diet (vitamins C and E, polyphenols, phytosterols, and plant extracts) in treatment of vanadium toxicity examined in animal and cell culture models. Additionally, possible direct and indirect mechanisms that could be involved in the beneficial activity of these antioxidants against vanadium have also been suggested. Studies identified in this review may help in the development of dietary strategies to improve protection of humans at high risk of vanadium toxicity.

## 2. Overview of Vanadium

**Figure 1:**
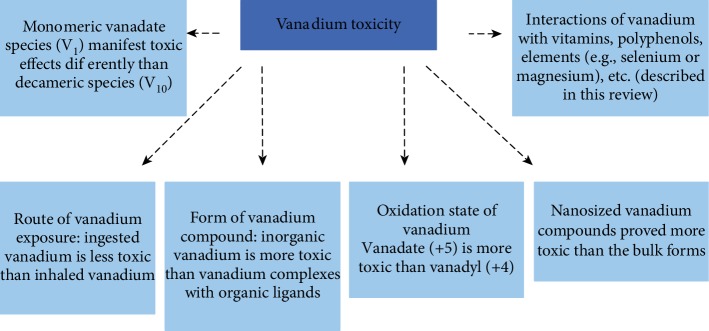
Factors affecting the toxicity of vanadium, according to References [13, 130, 131].

Vanadium occurs as a natural component of the earth crust (in various minerals, coal, and crude oil) and is released to the environment mainly due to human activities. The unique chemical and physical features of vanadium compounds make it an indispensable material in many industries. Its compounds are frequently used in the production of steel and titanium-aluminum alloys, as catalysts in the sulfuric acid manufacture, and in the production of pigments, inks, and varnishes [13, 14]. The latest use of vanadium involves green technologies and the production of vanadium-based redox flow batteries, which can store electricity produced from renewable sources such as wind or sun. These very efficient and increasingly popular energy storage systems have already been installed, e.g., in China, the USA, Germany, and Japan [15]. The industrial use of vanadium is on the increase and so is the release of vanadium to the environment [12]. Vanadium is one of the elements listed on the second drinking water contaminant candidate list (CCL-2) that was announced by the United States Environmental Protection Agency in 2005. This is a list of contaminants that are known or anticipated to occur in public water systems and may require future regulations [16]. Vanadium was reported to contribute to soil pollution. For example, soils from the stone coal smelting district in Hunan province of China had vanadium concentrations in the range from 168 to 1538 mg/kg, which substantially exceeded Canadian soil quality standards (130 mg/kg) [17]. Yang et al. [12] conducted research of the national soil pollution in China, and the results showed that 26.49% of soils were contaminated with vanadium in the southwest of China and 8.6% of the national soil pollution survey samples were contaminated with vanadium presenting a threat to the public and environment. Heavy oil combustion contributes to the release of vanadium as a component adhering to fine particulate matter (PM_2.5_) observed in large urban and industrial agglomerations such as New York City, the USA [18], and Jeddah, Saudi Arabia [19]. High groundwater concentrations of vanadium of natural geological sources have been noted in volcanic areas. For example, in some areas of Mt. Etna, groundwater vanadium content exceeded the Italian legal limit of 140 *μ*g/l; the consequently estimated daily intake of vanadium in children calculated in this study was in the range of 0.4-11 *μ*g/kg/bw, which was much higher than the estimated daily intakes of 0.09-0.34 *μ*g/kg/day reported in the literature [20]. Vanadium excess can be toxic and detrimental to human health like any other metal. For instance, occupational inhalation exposure to vanadium was found to induce, e.g., acute respiratory symptoms in boiler makers [21], DNA damage in blood cells of workers from a vanadium pentoxide factory [22], and altered neurobehavioral functions in Chinese workers [23]. In turn, environmental overexposures to vanadium oxides attached to fine particulate matter (PM_2.5_) were associated with, e.g., increased risk of respiratory symptoms in children of New York City [24] and a higher risk of cardiovascular and respiratory hospitalizations of older people in US counties [25]. Recently, urinary vanadium concentrations during pregnancy were positively associated with impaired fetal growth [26] and preterm or early-term delivery [27] in China. Association between the high level of trace elements (including vanadium) in the drinking water in the Mt. Etna volcanic area and the increased thyroid cancer incidence was suggested by Malandrino et al. [28]. A suicidal death after ingestion of an undetermined amount of ammonium vanadate has also been reported [29]. In addition, laboratory-based studies conducted in animal models or cell cultures found that vanadium exposure can induce a variety of toxic effects such as cardiovascular effects (e.g., vascular endothelial dysfunction [30] and arterial hypertension [31]), immune toxicity (e.g., damage to the spleen [32] and thymus [33]), neurotoxicity (e.g., hippocampal alterations [34] and memory loss [35]), developmental disturbances (e.g., increased embryolethality and skeletal defects [36]), and pulmonary toxicity [37]. It should be added that, besides the dose of vanadium and the route of vanadium exposure, many other factors such as the form of vanadium (inorganic versus organic forms) and interactions with other elements such as selenium [38, 39] or magnesium [40] can also influence vanadium toxicity (depicted in Figure 1).

Along with the studies of the toxic effects of vanadium, many investigators have been focused on the examination of potential medical applications of this mineral. These include antidiabetic (insulin-mimetic) actions, antiviral effects, and anticarcinogenic activity. Among these effects, the antidiabetic action of vanadium complexes with organic ligands has been very intensively studied since 1990s [41]. One of such compounds is bis(ethylmaltolato)oxidovanadium(IV) (BEOV), which entered into stage II clinical trials. However, due to kidney problems in some patients, BEOV as an antidiabetic agent could not progress to the next phase of research. Indeed, the risks associated with vanadium intoxication such as vanadium-induced reactive oxygen species generation, adverse effects on the immune system, and a risk of mutagenesis are listed among the arguments against the antidiabetic application of vanadium [42]. Domingo and Gomez [43] reviewed the results of past and recent human studies on vanadium in diabetes and concluded that the use of vanadium compounds in oral diabetes therapy is misplaced. Vanadium compounds have attracted interest of researchers as potential antitumor agents. For example, promising *in vitro* and *in vivo* anticancer activity was demonstrated for oxidovanadium(IV) complex with flavonoid chrysin [44, 45]. Vanadium as vanadyl sulfate has been used by weight training athletes as a nutritional supplement that can increase muscle mass [46]. The role of vanadium in muscle development has been emphasized to be associated with its insulin-mimetic properties and anabolic effects [47]. So far, however, human studies have failed to demonstrate significant effects of vanadium on the body composition and performance enhancement, and the use of vanadium as a sport nutrition supplement is not recommended [46]. Vanadium is also a well-known constituent of the most commercialized titanium alloy named Ti-6Al-4V, which has been widely used in the manufacture of biomedical implants such as artificial hip joints, knee joints, and dental implants due to its excellent physical and mechanical properties [48]. Again, however, the potential cytotoxicity of vanadium limits the medical value of the Ti-6Al-4V alloy [49]. Recently, for example, a case of systemic allergic dermatitis to vanadium has been reported in a patient following placement of a titanium alloy (Ti-6Al-4V) plate in the left foot [50]. Summing up, due to the intensive use of vanadium in industry and the vanadium environmental pollution often related with it as well as the popularity of vanadium-based dietary supplements and medicinal applications of vanadium compounds, increasing numbers of humans are likely to experience the exposure to vanadium compounds in the near future.

## 3. Metabolism and Vanadium Detoxification Modes

Vanadium enters the human body via the gastrointestinal tract or respiratory system. In the bloodstream, transferrin is the major serum protein of vanadium transport from blood into tissues [51]. Other serum proteins, i.e., albumin, hemoglobin, and immunoglobulin, and low-molecular ligands, e.g., lactate and citrate, can be involved in the blood transport of vanadium as well. From the blood, vanadium is transferred to different tissues such as the liver, kidney, heart, spleen, brain, and bones [52]. Final excretion of absorbed vanadium occurs through urine [53]. In the human body, vanadium can exist in oxidation state +5 (vanadate ions) or +4 (vanadyl cations). Cellular uptake of vanadium species proceeds via receptor-mediated endocytosis of vanadium-laden proteins (transferrin, albumin), phosphate or sulfate ion channels, or membrane citrate transporters [52]. Reductants, e.g., glutathione, ascorbic acid, or NADH, convert pentavalent vanadium to a tetravalent state (vanadyl), the latter being regarded as a predominant oxidation state of vanadium within the cell. Simultaneously, oxidants such as NAD+, O_2_, and O_2_^2-^ can oxidize vanadyl back to vanadate [54, 55].

Metabolic detoxification of vanadium possibly involves (1) reduction of vanadate to vanadyl by cellular reductants (mentioned above) and (2) complexation reactions during which vanadyl interacts with cellular agents such as reduced glutathione (GSH), an oxidized form of glutathione (GSSG), L-cysteine, and cystine forming stable, nonharmful complexes [56]. In addition, vanadium accumulates in bones by replacing bone phosphate in apatite Ca_5_(PO_4_)OH with vanadate [41]. The storage of vanadium in bones is also recognized as a potent detoxification mechanism of vanadium in animals [56].

Pharmacological treatment of vanadium poisoning is based on chelation therapy, which is the basic medical strategy for the treatment of acute and chronic intoxication with metals. Chelating agents are organic or inorganic compounds that can bind metal ions to form a stable, water-soluble complex with low toxicity, which are subsequently excreted from the body [3, 56]. With regard to the treatment of vanadium toxicity, calcium disodium ethylenediaminetetraacetate (CaNa_2_EDTA) has been found to enhance excretion of vanadium in calves. Simultaneously, however, the chelator did not protect against pathological damage caused by vanadium [57]. Another chelator, Tiron (4,5-dihydroxybenzene-1,3-disulfonate), was partly effective in reducing vanadium-induced behavioral toxicity in rats [58]. Other potential chelating antidotes for vanadium intoxication include desferrioxamine B, *meso*-2,3-disulfanylsuccinic acid (DMSA), and 2,3-disulfanylpropane-1-sulfonic acid (DMPS) [56]. However, besides benefits, the therapy with the drugs mentioned above may also induce side effects. For example, CaNa_2_EDTA was reported to cause nephrotoxicity, fever, headache, hepatotoxicity, and gastrointestinal symptoms. In addition, prolonged administration of CaNa_2_EDTA decreases the levels of essential metals. Treatment with DMSA and DMPS was reported to be associated with skin reactions, gastrointestinal discomfort, and elevated liver enzymes [3].

In contrast to the aforementioned chelating compounds, ascorbic acid was suggested to be a very effective and safe pharmacologic agent for the treatment of vanadium toxicity in humans [56]. Detoxification of vanadium by ascorbic acid mainly relies on ascorbic acid-mediated reduction of vanadate to vanadyl and its high capacity to scavenge reactive oxygen species. Furthermore, vanadyl was found to interact with oxidation products of ascorbic acid forming stable complexes, which may allow excretion of vanadium from the organism [59]. In addition, the results of studies from our group have shown that pyruvic acid could be another potential antidote for the treatment of vanadium toxicity [60]. The studies showed that this alpha-keto acid protected against vanadium-induced oxidative stress and cytotoxicity in a cell culture model. The mechanism of protection probably involves antioxidative effects of pyruvate, especially its ability to neutralize hydrogen peroxide, but still more research is required to elucidate this issue [60].

## 4. Role of Oxidative Stress in Vanadium-Induced Toxicity

Increased generation of reactive oxygen species (ROS) and oxidative stress play a predominant role in vanadium-induced cytotoxicity. For example, vanadate-induced cytotoxicity towards mouse epidermal JB6 cells [61] and monkey epithelial Ma104 cells [62] was demonstrated to be related with increased hydrogen peroxide (H_2_O_2_) formation. Vanadium-mediated formation of the hydroxyl radical (^·^OH) by activated human neutrophils was shown by ESR spectroscopy after *in vitro* exposure of these cells to vanadium in the +2, +3, and +4 valence states [63]. In an *in vivo* experimental study, significantly increased levels of hydroxyl radical and superoxide anion (O_2_^·-^) were detected in the cerebellum of sodium metavanadate-treated rats [64]. The vanadium-induced production of ROS occurs as a result of interconversion between V^4+^ species and V^5+^ species by the action of cellular oxidants (e.g., O_2_ and H_2_O_2_) and antioxidants within the cytoplasmic compartment, as described below. For example, the bioreduction of vanadate with NADPH in the presence of NADPH oxidase leads to the formation of vanadyl and superoxide radical. The superoxide is next decomposed by superoxide dismutase (SOD) to less toxic hydrogen peroxide and oxygen. In a Fenton-like reaction, vanadyl can be oxidized with hydrogen peroxide to vanadate with generation of highly reactive hydroxyl radicals [62]. The reaction of vanadate with superoxide anions leads to the formation of peroxovanadyl [V(+4)-OO^·^]. This radical can use hydrogen from NADPH and transform to vanadyl hydroperoxide, which in turn can decompose to vanadate and hydrogen peroxide via reaction with hydrogen [65]. Moreover, vanadium can directly affect the mitochondrial inner membrane, which subsequently may impair electron transfer between respiratory complexes causing generation of ROS in mitochondria [66]. ROS are implicated in mediating the deleterious actions of vanadium in cells and tissues through their reactions with cellular lipids, proteins, and nucleic acids. A reaction of lipids with ROS (lipid peroxidation) has a chain character and leads to oxidative degeneration of phospholipids in cell membranes. The two major consequences of lipid peroxidation include changes in membrane biophysical properties (e.g., increased permeability and altered fluidity) and generation of lipid peroxidation end products, many of which are toxic to cells [67]. Various studies indicate that vanadium-induced lipid peroxidation is implicated in toxic effects of vanadium compounds on the liver [68, 69], kidney [70], and brain [71]. In addition, vanadium-mediated oxidative stress manifested by protein oxidation or DNA oxidation has also been observed in vanadium-exposed animals [71] and humans [22], respectively. ROS and oxidative stress have also been reported to contribute to vanadium-induced pulmonary inflammation [72], neurotoxicity [73], and carcinogenic-related effects [74].

## 5. Dietary Antioxidants in the Prevention of Vanadium Toxicity

It is well known that many edible plants are the main source of natural compounds acting as exogenous antioxidants. Exogenous antioxidants cannot be produced in the body and therefore must be provided through daily nutrition. They reinforce our intrinsic antioxidant system in the protection of the organism against reactive oxygen species-mediated injuries [75]. As shown below in this review, research studies indicate that vanadium toxicity, which is strongly associated with prooxidant mechanisms, can be efficiently reduced or alleviated by dietary and plant-derived antioxidants. The details of the studies on the protective effects of exogenous antioxidants against vanadium adverse actions are presented in Table 1.

### 5.1. Vitamins C and E

Very early studies already explored the efficiency of vitamin C (ascorbic acid, ascorbate) in the prevention and treatment of vanadium toxicity. For example, a study by Jones and Basinger [76] found that vitamin C was effective against acute vanadate and vanadyl intoxication in mice. Vitamin C, similar to a chelating agent Tiron, was reported to increase urinary excretion of vanadium in mice following acute exposure to vanadyl sulfate [77]. In contrast, other early studies in rats failed to show that vitamin C could influence urinary elimination or tissue concentration of vanadium [78, 79]. More recently, very few studies investigated the interactions of vitamin C and vanadium. In one published study, vitamin C partly enhanced the serum antioxidant status and egg quality in ammonium metavanadate-intoxicated hens [80].

Some studies focused on the role of vitamin E (*α*-tocopherol) in the treatment of vanadium toxicity. For example, a study by Chandra et al. [81] provided *in vivo* evidence that vitamin E acetate decreased sodium metavanadate-induced oxidative stress and histopathological changes in the testes of rats. Furthermore, vitamin E was demonstrated to exhibit protective activity against sodium metavanadate-mediated neurotoxicity in rat pups [82]. In this study, vitamin E increased performance in neurobehavioral tests (though not statistically significantly) and decreased reactive astrogliosis in brain tissue of vanadium-treated animals. Both vitamins C and E exhibited protective activity against vanadium pentoxide-induced genotoxicity measured using a micronucleus assay in mouse polychromatic erythrocytes [83].

The antioxidant effect of vitamins E and C is related to their high reactivity as hydrogen (vitamin E) or electron donors (vitamin C) to free radical oxidants, which prevent oxidative damage to cells and tissues, as described below. Vitamin E is a fat-soluble vitamin; in the form of *α*-tocopherol, it is a major antioxidant located within biological membranes playing a role in protecting from lipid peroxidation. *α*-Tocopherol breaks the chain reactions of lipid peroxidation through the mechanism of donation of a hydrogen atom from its phenolic hydroxyl group to lipid peroxyl radical resulting in the formation of stable lipid hydroperoxide and unreactive tocopheroxyl radicals [84]. Vitamin C readily donates electrons to oxygen-related radicals (e.g., hydroxyl and peroxyl radicals), sulfur radicals, and nitrogen-oxygen radicals [85]. In addition, vitamin C is able to regenerate *α*-tocopherol by reducing tocopheroxyl radical to its original form (reduced *α*-tocopherol) [86].

### 5.2. Polyphenols

Polyphenols (also known as phenolics) are the most numerous and highly diverse group of phytochemicals. They are divided into four subclasses (flavonoids, stilbenes, lignans, and phenolic acids) based on the number of phenolic rings and structural elements that bind these rings to one another. In plants, polyphenols serve a protective role, defending the plant against ultraviolet radiation, cold temperatures, droughts, and incoming pathogens [87, 88]. In humans, polyphenol-rich diet has been associated with the prevention of diseases like certain cancers, cardiovascular diseases, type 2 diabetes, and neurodegenerative disorders [89]. Studies suggest that they can also provide protection against cadmium and lead toxicity [90]. These health-promoting effects of polyphenols are explained by their antioxidant, anti-inflammatory, and metal-chelating actions [88, 91].

As described in Table 1, some very recent studies have shown that polyphenols can be protective against vanadium toxicity in animals. Accordingly, tea polyphenols (a mixture of catechin, epigallocatechin gallate, and caffeine) alleviated vanadium-induced toxic effects on liver antioxidant enzymes [92] and vanadium-mediated epithelial cell apoptosis of the duodenum [93] in hens. Other investigations have focused on a specific polyphenolic compound, namely, epigallocatechin gallate (EGCG). This polyphenol, classified to the group of flavonoids, is the most abundant catechin from green tea infusion with high antioxidant activity [94]. Studies have shown that administration of this phenolic compound to animals protected against oxidative stress and histopathological changes induced by ammonium metavanadate in rat kidneys [95]. Epigallocatechin gallate had also a protective effect on vanadium-induced oxidative stress in the uterus of hens [96]. There are different mechanisms by which polyphenols can exert their antioxidant actions. These include (1) direct radical-scavenging activity by H-atom transfer or by electron transfer from polyphenol to an unstable free radical [97], (2) chelation of metal ions such as iron or copper thereby preventing them from the production of free radicals [98], (3) inhibition of enzymes that can generate radicals, e.g., cytochrome P_450_ isoforms, lipooxygenases, and cyclooxygenases, and (4) synergistic interactive effects of polyphenols with other antioxidants such as phytosterols [99, 100]. However, which of these mechanisms is responsible for the antioxidant effect of phenolics against vanadium-induced toxicity remains to be elucidated.

In addition, polyphenolic compounds (and other phytochemicals) may prove beneficial for the treatment of vanadium toxicity through their ability to activate Nrf2 signaling. Nuclear factor erythroid 2-related factor 2 (Nrf2) is a transcription factor, which upon activation translocates to the nucleus and binds to antioxidant response element (ARE) sequences inducing expression of different cytoprotective enzymes. Nrf2-mediated enzymes include antioxidant enzymes such as catalase (CAT), superoxide dismutase (SOD), glutathione peroxidase (GPx), and heme oxygenase-1 (HO-1) as well as enzymes responsible for the synthesis and regeneration of glutathione (GSH) such as glutamate cysteine ligase (GCL), glutathione synthetase (GSS), and glutathione reductase (GR) [101]. Thus, activated Nrf2 can significantly increase the antioxidant response in cells to fight oxidative stress associated with vanadium toxicity. Furthermore, vanadium was shown *in vitro* to exert a negative effect on the Nrf2 pathway by inhibiting both the translocation of the Nrf2 factor to the nucleus and the expression of the Nrf2 inducible enzyme NAD(P)H:quinone oxidoreductase 1 (NQO-1) in Hepa 1c1c7 cells [102]. Very recent *in vivo* data provided evidence that dietary vanadium downregulated Nrf2 and heme oxygenase-1 expression in the uterus of hens and coexposure to epigallocatechin gallate prevented this effect and additionally markedly reduced the vanadium-induced uterine oxidative stress [96]. The mechanisms of Nrf2 activation by epigallocatechin gallate were suggested to involve phosphorylation of Nrf2 serine/threonine residues by protein kinase P38-MAPK, which could upregulate Nrf2 nuclear translocation and subsequent ARE binding [96].

Moreover, some cytoprotective actions of polyphenols may be related to their positive effects on gut microflora. It is suggested that polyphenols through not yet known mechanisms promote beneficial intestinal flora (e.g., bifidobacteria) and inhibit invasive species [89]. Intestinal microbes produce short-chain fatty acids, including butyrate, acetate, and propionate, during fermentation of dietary fiber. These short-chain fatty acids, and butyrate in particular, have been linked with beneficial effects on the metabolism of epithelial cells and preventive effects against colonic cancer [103]. A recent study showed that dietary supplementation of vanadium reduced cecum butyrate acid content and tea polyphenols prevented this reduction in hens [93]. The authors suggested that, by increasing the butyrate content, polyphenols protected duodenal cells from vanadium-induced apoptosis.

### 5.3. Phytosterols

Phytosterols are a group of steroid compounds present in plant food with the highest amount found in vegetable oils [104]. In plants, their function is to stabilize the phospholipid bilayer of cell membranes. In functional (and structural) terms, they are analogous with cholesterol in humans. Dietary intake of plant sterols by humans has been shown to block both biliary and dietary absorption of cholesterol in the intestine, which helps to lower blood cholesterol levels [105]. In addition, phytosterols have been associated with other beneficial health effects in humans and animals such as reduced risk of heart diseases, anti-inflammatory activities, and prevention of certain cancers [104].

Although limited in number, there are studies evaluating the effects of phytosterols on vanadium-induced toxicity, as described below. The oral administration of stigmasterol and *β*-sitosterol significantly attenuated neurobehavioral impairments such as deficits in exploration, learning and memory disabilities, and decreased motor coordination, which were induced by sodium metavanadate in mice [106, 107]. Additionally, in these studies, the two sterols mentioned above exerted an inhibitory effect on hydrogen peroxide generation and lipid peroxidation and improved the activities of superoxide dismutase and catalase in brain tissue of vanadium-exposed mice. Therefore, the authors proposed that the neuroprotective effects of stigmasterol and *β*-sitosterol could be associated with reduced levels of oxidative stress [106, 107]. The exact mechanism of the antioxidant activity of phytosterols is not well understood. Nevertheless, some *in vitro* studies were undertaken to clarify this issue. For example, it has been found that the extract of the Asian shrub *Aglaia oligophylla*, which contained *β*-sitosterol and stigmasterol in addition to oligophyllic acid, possessed high antioxidant activity measured by cupric reducing antioxidant capacity (CUPRAC) and ferric reducing antioxidant power (FRAP) assays [108]. The antioxidant effect of *β*-sitosterol and stigmasterol observed in this study has been attributed to their ability to donate electrons from their hydroxyl or carboxyl groups directly to the free radical thus neutralizing it [108]. Additionally, another *in vitro* study suggested that *β*-sitosterol prevented superoxide anion and hydrogen peroxide production by RAW 264.7 macrophages stimulated by phorbol myristate acetate (PMA) due to enhancement of endogenous intracellular antioxidant defenses [109]. In line with this, the study with the same cell culture model as above found that *β*-sitosterol recovered glutathione (GSH) levels and the GSH/total glutathione ratio and enhanced the activities of antioxidant enzymes, probably through the estrogen receptor/phosphatidylinositol 3-kinase (PI3-kinase) pathway [110]. More recently, *in vivo* research implicated the Nrf2 transcription factor in beneficial effects of *β*-sitosterol against N-diethylnitrosamine- and ferric nitrilotriacetate-induced nephrotoxicity in rats [111]. So far, however, there are no data available that could indicate which exact mechanism could be involved in the beneficial effects of phytosterols against vanadium intoxication.

### 5.4. Plant Extracts

Some studies were designed to explore the protective effects of formulations obtained from parts of plants, e.g., leaves or flowers, rather than effects of single isolated components against vanadium-induced toxicity. For example, the results reported that *Malva sylvestris* (prepared as decoction of leaves and flowers) alleviated ammonium metavanadate-induced nephrotoxic effects in rats measured by lipid peroxidation, antioxidant enzyme activities, and histopathological changes [70]. The authors suggested that the antioxidant activities of flavonoids were mostly involved in these beneficial effects. In addition, *M. sylvestris*, particularly its leaves, is also rich in carotenoids and tocopherols and the flowers of this plant exhibited high content of ascorbic acid. All these components are well known for their radical-scavenging activity [112]. The essential oil of the sage *Salvia officinalis* exerted a similar antioxidant effect against ammonium metavanadate-induced renal toxicity [113] as that shown by *M. sylvestris* extract. The sage essential oil contains a diverse group of bioactive phytochemicals with antioxidant, anti-inflammatory, and nephroprotective properties like camphor, *α*-pinene, *α*-thujene, carveol, or *α*-terpineol, which were assumed to contribute to the protective activity of *S. officinalis* [113, 114]. The leaf extracts of plants such as *Moringa oleifera* [115] and *Grewia carpinifolia* [116] were also reported to have beneficial effects against vanadium-induced neurotoxicity, and green tea (*Camellia sinensis*) [117] was active against vanadium-induced renal, hepatic, and testicular lipid peroxidation in rodents. The protective effect of green tea could be probably attributed to green tea polyphenols, among which catechins (epicatechin, epigallocatechin, epicatechin gallate, and epigallocatechin gallate) are the most abundant tea polyphenols showing many health-promoting benefits including antioxidant (discussed in the earlier section), anti-inflammatory, and antimutagenic effects [118]. In turn, *M. oleifera* is a well-known herbal plant in Africa and Asia, whose leaves contain a diverse mixture of bioactive phytochemicals such as phenolic acids (caffeic acid, gallic acid), flavonoids (e.g., quercetin, kaempferol, and catechins), saponins, tannins, vitamins, and carotenoids. They have been reported to underlie the health protective potential of Moringa [119] and may contribute to the alleviation of vanadium-mediated toxicity.

### 5.5. Other Possible Dietary Supplements against Vanadium

Some mineral components have also been reported to reduce toxicity associated with vanadium exposure. For example, the essential metal zinc was found to reduce the vanadium-induced DNA damage (comet assay) in melanocytes *in vitro* [120]. Oral supplementation of zinc sulfate prevented lipid peroxidation and normalized the activities of antioxidant enzymes in the testis of sodium metavanadate-exposed rats [121]. As shown by the *in vitro* study of Bay et al. [122], zinc has the ability to reduce the levels of hydroxyl free radicals generated by vanadium in a Fenton-like reaction and prevent a decline in glutathione levels, which may constitute mechanisms through which zinc exhibits its protective effects against vanadium injury. Another element, i.e., magnesium, has shown a protective potential against vanadium as well. As reviewed by Ścibior [40], when given orally during vanadium intoxication in rats, magnesium caused the following effects: it reduced the level of vanadium in erythrocytes and whole blood, limited accumulation of vanadium in kidney and the cerebral hemisphere, decreased lipid peroxidation in the liver, and prevented the decrease in glutathione transferase activity in erythrocytes. Lastly, selenium either alone or in conjunction with a chelating agent Tiron exhibited protective effects against vanadium-induced injury in rats [38, 123]. However, converse results were obtained in mammalian cells *in vitro* (CHO-K1 cells) where vanadyl-induced cytotoxicity was increased in the presence of low doses of selenite (0.5 and 1 *μ*M) [39]. The contradictory results on vanadium and selenium interactions appear to confirm the well-known “two-faced” character of selenium, most probably caused by a very narrow range in selenium concentrations between necessity and toxicity. This certainly raises concerns over the use of selenium (especially as selenite) as a potential antidote against vanadium.

## 6. Conclusions

**Figure 2:**
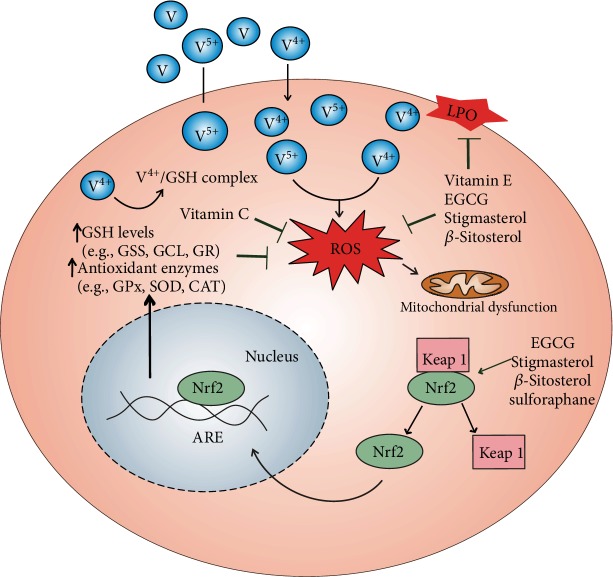
Scheme showing the putative cellular sites of action targeted by some dietary antioxidants during vanadium intoxication. Whether polyphenols (e.g., EGCG) and phytosterols (e.g., stigmasterol and *β*-sitosterol) act as direct antioxidants (through their scavenging activity) or/and indirect antioxidants (e.g., by inducing Nrf2 binding to ARE) remains to be confirmed. Abbreviations: ARE: antioxidant response element; CAT: catalase; EGCG: epigallocatechin gallate; GCL: glutamate cysteine ligase; GPx: glutathione peroxidase; GR: glutathione reductase; GSS: glutathione synthetase; Keap1: Kelch-like ECH-associated protein 1; LPO: lipid peroxidation; Nrf2: nuclear factor (erythroid-derived 2)-like 2; ROS: reactive oxygen species; SOD: superoxide dismutase; V^5+^: pentavalent vanadium; V^4+^: tetravalent vanadium; 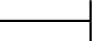: inhibition; 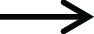: stimulation.

This review provides an updated overview of the role of different dietary-derived antioxidants in vanadium-induced toxicity. In general, the studies show the therapeutic protective effects of vitamins C and E, tea polyphenols, phytosterols, and some plant extracts against vanadium. As expected, the beneficial action of these natural compounds is based on their ability to reduce vanadium-mediated oxidative stress (some possible cellular sites of protection are summarized in Figure 2). The mechanism of action of vitamins C and E probably includes direct removal of reactive oxygen species from the intracellular compartment (by hydrophilic vitamin C) and within the cell membrane (by lipid-soluble vitamin E). As mentioned in this review, polyphenols and phytosterols have also the ability to neutralize reactive oxygen species directly via donating electrons or hydrogen atoms from their hydroxyl (or carboxyl) groups. However, some authors suggest that polyphenols perform their antioxidant activity through other mechanisms, e.g., induction of antioxidant and phase II enzymes, which are indirect modes of antioxidant activity rather than acting through direct reactive oxygen species scavenging mechanisms. The reason for that would be the lower reduction potential and bioavailability of polyphenols compared to endogenous antioxidants [124]. So far, only one study has assessed the indirect antioxidant mechanism of polyphenols against vanadium showing increased Nrf2 and HO-1 expression as a protective mode of epigallocatechin gallate action in vivo [96]. Therefore, the mechanisms of action of exogenous antioxidants in prevention of vanadium toxicity remain to be further clarified. In addition, beneficial effects of extracts from medicinal plants such as *Moringa oleifera* or *Malva sylvestris* against vanadium have also received attention. The major advantage of plant extracts is their content of a mixture of different phytochemicals and nutrients which, via synergistic/additive interactions, are suggested to be more health effective than isolated phytochemicals [75, 125].

In conclusion, although the investigations cited in this review show that supplementation with dietary antioxidants has beneficial effects on vanadium poisoning, further studies have to be conducted to draw more definitive statements. The following points have been identified as topics for future research:

1. Still, more studies are needed on the role of vitamins C and E in the toxicology of vanadium. The relatively low cost and wide therapeutic window (especially for vitamin C) of these nutrients make them attractive antidotes against vanadium poisoning
2. The precise mechanism of the activity of polyphenols and phytosterols against vanadium should be explored
3. Therapy with plant extracts containing a mixture of different phytochemicals could be an interesting alternative to single compound treatment

## Figures and Tables

**Table 1:** Summary of the effects of dietary antioxidants on vanadium toxicity in animal and cell culture models.

Vanadium compounds	Dietary antioxidants	Animal/cell culture model	Effects compared to vanadium-treated animals or cells	Ref.
Vanadium-vitamins				
NH_4_VO_3_ 5 and 10 mg/kg diet	Vitamin C 50 and 100 mg/kg diet	L hens	↑ Egg quality; ↑ serum SOD activity; ↓ serum LPO	[80]
V_2_O_5_ 40 mg/kg, ip	Vitamin C (100 mg/kg, ip) or vitamin E (20 mg/kg by gavage)	Hsd:ICR mice (♂)	↓ Micronucleated polychromatic erythrocytes	[83]
NaVO_3_ 0.4 mg V/kg bw, ip	Vitamin E acetate 50 and 100 mg/kg bw, orally	SD rats (♂)	↑ Reproductive organ weight; ↑ sperm number and morphology; ↑ testicular steroidogenic enzyme activities; ↑ serum testosterone, LH and FSH levels; ↑ testicular SOD, CAT activities; ↓ testicular LPO; ↓ testicular histopathological changes	[81]
NaVO_3_ 3 mg/kg bw, ip	Vitamin E 500 mg/kg bw, orally	Nursing albino rats	In pups coexposed to vitamin E and vanadium through lactation: ↑ body weight gain, ↑ brain weight, ↓ reactive astrogliosis, ↑ locomotor and exploratory activity, ↑ hanging latency	[82]
NaVO_3_ 3 mg/kg bw, ip (pubs)	Vitamin E 500 mg, orally (dams)	W rats (dams and pubs)	In vanadium-treated pups exposed to vitamin E through lactation: ameliorated histopathological changes in the testes, lungs, and liver	[126]
Vanadium-polyphenols (flavanols)				
NH_4_VO_3_ 5, 10, and 15 mg V/kg diet	Tea polyphenols 600 and 1000 mg/kg diet	L hens	↑ Hepatic GST and GPx activities; ↑ production and egg quality	[92]
NH_4_VO_3_ 10 mg V/kg diet	Tea polyphenols 600 and 1000 mg/kg diet	L hens	↓ Intestinal microflora diversity; ↓ duodenal cell apoptosis; ↑ cecum butyrate acid content	[93]
NH_4_VO_3_ 5 mg/kg bw, ip	Epigallocatechin gallate 5 mg/kg bw, ip	W rats (♂)	↓ Renal LPO; ↑ renal CAT, SOD, and GPx activities; ↑ serum vitamin E and A levels; ↓ histopathological changes in the kidneys	[95]
NH_4_VO_3_ 10 mg/kg diet	Epigallocatechin gallate 130 mg/kg diet	L hens	↑ Eggshell color; ↑ protoporphyrin IX content (in eggshell); ↓ uterine LPO; ↑ uterine GST activity; ↑ Nrf2 and HO-1 gene and protein level (in uterus); ↑ phospho-P38 MAPK protein level (in uterus)	[96]
Vanadium-polyphenols (flavonones)				
NaVO_3_ 1 mg/kg bw, ip	Glucosyl hesperidin 25 and 50 mg/kg bw, orally	SD rats (♂)	↑ Reproductive organ weight; ↑ sperm count, motility, and morphology; ↓ sperm DNA fragmentation; ↑ serum testosterone levels; ↓ testicular LPO; ↑ testicular SOD and CAT activities; ↓ testicular histopathological changes	[127]
Vanadium-polyphenols (stilbenes)				
NH_4_VO_3_ 5 mg/kg bw, ip	Resveratrol 50 mg/kg, orally	SD rats (♂)	↑ Body weight gain; ↓ blood urea nitrogen and creatinine level; ↑ renal SOD activity; no effects of resveratrol on vanadium-induced histopathological changes in the kidneys	[128]
Vanadium-phytosterols				
NaVO_3_ 3 mg/kg, ip	Stigmasterol 100 *μ*g, orally	BALB/c mice (♂)	↓ Hippocampal LPO and H_2_O_2_ levels; ↑ hippocampal SOD and CAT activities; ↑ learning and memory; ↑ locomotor and exploratory activity; ↑ hanging latency; ↓ damage to myelin sheaths, ↑ MBP expression	[106]
NaVO_3_ 3 mg/kg, ip	*β*-Sitosterol 100 *μ*g, orally	BALB/c mice (♂)	↑ Learning and memory; ↑ locomotor and exploratory activity; ↑ hanging latency; ↓ brain LPO and H_2_O_2_ levels; ↓ damage to myelin sheaths; ↑ MBP expression; ↑ SOD, CAT activities and GSH level in the brain	[107]
Vanadium-organosulfur compounds (isothiocyanates)				
VOSO_4_ 34.4 and 68.8 *μ*M	R-sulforaphane 5 *μ*M	HepG2, Caco-2, and Vero cells	↓ ROS; ↓ mitochondrial depolarization; ↑ lysosomal integrity; ↓ DNA damage (comet assay); ↓ cell death	[129]
Vanadium-plant extracts				
NaVO_3_ 100 and 200 *μ*M	*Moringa oleifera* leaf extract 0.063 mg/well, 0.01 and 0.02 mg/ml	Mouse hippocampal H22 cells	↓ Superoxide levels; ↓ DNA damage (comet assay)	[115]
NaVO_3_ 3 mg/kg, ip	*Grewia carpinifolia* leaf extract 200 mg/kg, orally	Swiss mice (♂)	↑ Locomotor and exploratory activity; ↑ hanging latency; ↑ motor coordination	[116]
NH_4_VO_3_ 5 mg/kg bw, ip	Green tea *Camellia sinensis* decoction 66 g of leaves/l, as a drink	W rats (♂)	↓ LPO in the kidney, liver, and testes; ↑ vitamins E and A in serum; ↓ CAT and SOD activities in erythrocytes	[117]
NH_4_VO_3_ 60 mg/kg bw (drinking water)	*Malva sylvestris* decoction 0.2 g of dry plant/kg bw	W rats (♂)	↓ Renal LPO, CAT, SOD, and GPx activities; ↓ histopathological changes in the kidneys	[70]
NH_4_VO_3_ 5 mg/kg bw, ip	Essential oil of *Salvia officinalis* 15 mg/kg bw, orally	W rats (♂)	↓ Plasma renal markers (creatinine, urea, uric acid, and LDH); ↓ renal LPO and protein carbonyls; normalized CAT, SOD, and GPx activities in the kidney; ↓ renal histopathological changes	[113]

Abbreviations: bw: body weigh; CAT: catalase; FSH: follicle-stimulating hormone; GPx: glutathione peroxidase; GSH: reduced glutathione; GST: glutathione S-transferase; H_2_O_2_: hydrogen peroxide; HO-1: heme oxygenase; ip: intraperitoneal; LDH: lactate dehydrogenase; LH: luteinizing hormone; L hen: Lohmann hen; LPO: lipid peroxidation; MAPK: mitogen-activated protein kinases; MBP: myelin basic protein; NaVO_3_: sodium metavanadate; NH_4_VO_3_: ammonium metavanadate; Nrf2: nuclear factor erythroid 2-related factor 2; ROS: reactive oxygen species; SD rat: Sprague Dawley rat; SOD: superoxide dismutase; V: vanadium; V_2_O_5_: vanadium pentoxide; W rats: Wistar rats; ↑: increased; ↓: decreased.
